# Valorization of Seaweed Wracks: Inclusion as Additive in Diets for Grass Carp (*Ctenopharyngodon idella*)

**DOI:** 10.1155/2022/6992682

**Published:** 2022-12-05

**Authors:** Ana Galindo, Covadonga Rodríguez, Diana B. Reis, Manuel Marrero, Nieves G. Acosta, Maria Carmo Barreto, Ignacio A. Jiménez, Jaime de Urioste, Marianna Venuleo, José A. Pérez

**Affiliations:** ^1^Departamento de Biología Animal, Edafología y Geología, Universidad de La Laguna, Avenida Astrofísico Francisco Sánchez s/n, San Cristobal de La Laguna, 38206 Tenerife, Spain; ^2^cE3c—Centre for Ecology, Evolution and Environmental Changes/Azorean Biodiversity Group and Faculty of Sciences and Technology, University of Azores, 9500-321 Ponta Delgada, Portugal; ^3^Instituto Universitario de Bio-Orgánica Antonio González, Departamento de Química Orgánica, Universidad de La Laguna, Avenida Astrofísico Francisco Sánchez 2, San Cristobal de La Laguna, 38206 Tenerife, Spain; ^4^Centro de Investigación y Conservación de la Biodiversidad, Fundación Neotrópico, C/Piñonero 9, Barranco Grande, 38311 Tenerife, Spain; ^5^Departamento de Biotecnología, División de Investigación y Desarrollo Tecnológico, Instituto Tecnológico de Canarias, Playa de Pozo Izquierdo s/n, 35119 Santa Lucía de Tirajana, Gran Canaria, Spain

## Abstract

Macroalgae have been recently described as a potential ingredient for aquafeeds, exerting several physiological benefits. Grass carp (*Ctenopharyngodon idella*) is a freshwater species, which has been the major fish species produced in the world in the last years. In order to determine the potential use of macroalgal wracks in fish feeding, *C. idella* juveniles were fed with an extruded commercial diet (CD) or the CD supplemented with 7% of a wind dried-powder (1 mm) from either a multispecific macroalgal wrack (CD + MU7) or a monospecific macroalgal wrack (CD + MO7) obtained from Gran Canaria island (Spain) coasts. After 100 days of feeding, survival, fish weight, and body indexes were determined, and muscle, liver, and digestive tract samples were collected. The total antioxidant capacity of macroalgal wracks was analyzed by assesing the antioxidant defense response and digestive enzymes activity in fish. Finally, muscle proximate composition, lipid classes (LC), and fatty acid (FA) profiles were also studied. Our results suggest that dietary inclusion of macroalgal wracks does not have negative effects on growth, proximate, and lipid composition, antioxidative status, or digestive capacity of *C. idella*. In fact, both macroalgal wracks caused a general lower fat deposition, and the multispecific wrack enhanced catalase activity in the liver.

## 1. Introduction

Fish meal and fish oil have traditionally been used as major ingredients in commercial aquafeeds as the most important sources of protein, amino acids, fatty acids (FA), minerals, and energy [1, 2] for carnivorous species. However, the fluctuating availability of marine ingredients, their sustained price rise, and the increment of global aquaculture production have driven the search for more sustainable alternatives [3].

Lipids, mainly formed by FA, are the primary organic components of fish, together with proteins. C18 polyunsaturated fatty acids (PUFA) such as linoleic acid (18:2n-6, LA) and *α*-linolenic acid (18:3n-3, LNA) are essential nutrients for vertebrates, and metabolic precursors of the physiologically important long-chain PUFA (LC-PUFA), particularly arachidonic acid (20:4n-6, ARA), eicosapentaenoic acid (20:5n-3, EPA), and docosahexaenoic acid (22:6n-3, DHA) [4]. LC-PUFA are involved in key physiological roles including transcription regulation, cell signaling, and cellular membrane structure [4]. Although the use of ingredients from terrestrial plants is one of the most common alternatives to fish meal and fish oil in aquafeed formulation, they are deficient in LC-PUFA, reducing the contribution of essential FA to fish flesh [5]. Additionally, crop-plant-derived protein sources have low digestibility and supply scarce amounts of some essential amino acids [6]. On the contrary, algae have been proposed as suitable alternative sources of lipids and proteins for farmed fish due to their high nutritional quality and balanced composition, high production rates, and potential availability [1, 6].

The inclusion of micro- and macroalgae in aquafeeds has been recently studied in both freshwater and marine fish species [1, 7, 8], being believed that fish response to dietary algal inclusion is dose-dependent and species-specific [6]. Small dietary amounts of algae (2.5-10%) produced positive effects in fish growth performance, feed efficiency, lipid metabolism, body composition, stress response, liver function, and disease resistance, among others, in so far species studied [1, 6]. However, a high *Pterocladia* sp. and *Ulva rigida* inclusion (>10%) led to poor growth and reduced feed efficiency in gilthead seabream, *Sparus aurata* [9], and Nile tilapia, *Oreochromis niloticus* [10], respectively. This detriment in growth was attributed to the presence of antinutrients such as saponins, tannins, phytic acid, and protease and amylase inhibitors [10], which are present in the vegetative tissues of terrestrial plants and that have been also suggested in algae [6, 10].

All aerobic organisms, including fish, are susceptible to reactive oxygen species (ROS) stress [11]. Fish tissues contain large amounts of PUFA, which are essential for membrane structure but are highly vulnerable to be oxidized. Consequently, fish should have an effective antioxidant system to prevent PUFA oxidation [11]. To mitigate damage caused by ROS, fish as other animals have developed antioxidant defenses, including a number of enzymes involved in preserving redox homeostasis such as superoxide dismutase (SOD), catalase (CAT), glutathione-S-transferase (GST), or glutathione reductase (GR) but also antioxidant molecules like carotenoids, vitamins, or peptides [11]. Either biotic or abiotic factors may increase or decrease the antioxidative responsive mechanisms in fish. Thus, dietary lipid level, including high PUFA levels, vitamins, minerals, and the type of starch, between others, have been associated with oxidative stress in fish. Antioxidative response of fish may also depend on the dietary supply of antioxidants such as vitamin E, which is the main soluble antioxidant present in animals [11]. In this sense, polysaccharides and fucoxanthin (a marine carotenoid found in brown macroalgae and silicified microalgae, i.e., diatoms) have been reported as important mediators in lipid metabolism and are being increasingly studied in human and animal nutrition [6].

*In vitro* studies have evidenced antioxidant properties of seaweed [12], and even algae consumption has been related to an increase in the endogenous antioxidant enzymes SOD and CAT activities in some mammals *in vivo* [13]. Although there is an emerging interest in determining the role of dietary seaweed supplementation on antioxidant and immune responses in fish [14], these studies are still scarce [12, 14–16]. Previous investigations suggested that feed supplemented with macroalgae may moderate stress responses and thus improve vitality, illness resistance [13, 15], and flesh quality of fish [16], which are important parameters for the aquaculture industry [14].

Strandings of macroalgal wracks that regularly detach from offshore seaweed beds and then accumulate on the coasts play a key role in beach ecosystems, preventing coastal erosion and acting as both a source of organic matter and a substrate for several invertebrates [17]. However, this clumping natural biomass is often interpreted as an indicator of beach poor quality by bathers and thought to compromise the aesthetics of the beach, as well as charged to cause unpleasant smell after decomposing. Thus, algae accumulations are usually removed and dumped in local landfills, causing an increased pressure on the handling and management of beach wracks [18]. New uses for this biological biomass are being currently evaluated [19–21], trying to reduce the environmental and economic impact linked to its managing compared to its simple disposal.

Several studies have been conducted to reduce fish meal in diets for grass carp *Ctenopharyngodon idella* [22–24], including that of Salama et al. [25] where a 25% dietary inclusion of the microalgae *Arthrospira platensis* (Spirulina) did not significantly affect fish performance and whole body composition. However, the inclusion of macroalgal wracks for this purpose has not yet been addressed. Thus, the present study was undertaken to evaluate the use of Macaronesian macroalgal wracks as a feasible supplement in aquafeeds for *C. idella* from an ecophysiological perspective.

## 2. Material and Methods

The ULL Ethical Committee (CEIBA, Comité de Ética de la Investigación y Bienestar Animal) approved all experimental procedures (CEIBA2015-0165) in accordance with Spanish Royal Decree 53/2013, of 1^st^ February on the protection of animals used for experimentation or other scientific purposes.

### 2.1. Macroalgal Wrack Collection and Pretreatment

Macroalgal wracks were removed with a bulldozer-like machine from Las Canteras beach (28°08′24^″^N, 15°26′15^″^W) in Gran Canaria (Spain) as part of the ordinary beach management procedure operated by local public administrations. Random samples of this biomass with a minimum weight of 20 kg representing at least 1% of the total biomass collected in each cleaning event were separated from sand, washed with seawater, and dried by the action of continuous natural wind in the shadow. Subsamples of wet biomass were used for taxonomic identification. After 24 h, dried samples were crushed and ground to a fine powder (1 mm) with a rotor beater mill (SR 30; Retsch GmbH, Haan, Germany) and stored at room temperature in the dark [20].

### 2.2. Experimental Conditions

Grass carp (*C. idella*) juveniles were obtained from Pisciber Bio Secure Fishes, S.L. (Terrasa, Barcelona, Spain). Before the beginning of the trial, fish were maintained in the rearing system and fed with the control diet for 3 weeks to acclimatize to the experimental conditions. The experiment was carried out in 1 m^3^ polyethylene tanks under recirculating aquaculture system (RAS) equipped with an Eheim Biopower 240 biofilter and a recirculation pump Eheim compact+3000 (Eheim GmbH & Co. KG, Deizisau, Germany) with a water flow rate of 1000 L h^−1^. In the manufacturing of diets, a basal extruded commercial diet (TI-3 Tilapia Skretting) with protein and lipid contents of 35.63 and 9.19% of DW, respectively, was minced, supplemented or not with the macroalgal wrack, and repelletized to avoid texture and palatability differences among diets. Along the experimental period, fish were daily fed with a 3-5% of their total biomass, three times a day. Thus, 207 *C. idella* juveniles (initial weight 33.53 ± 8.02 g) were randomly distributed into 9 experimental tanks (by triplicates, 23 individuals in each tank) and fed the commercial diet (CD, control group) or the same diet supplemented with either a 7% of multispecific (MU) macroalgal wrack (33.8% *Asparagopsis taxiformis*, 28.6% *Lobophora* sp., 22.6% *Dictyota* sp., 14.5% *Cymopolia barbata*, and 0.5% *Laurencia* sp.) (experimental treatment 1, CD + MU7) or a 7% of monospecific (MO) macroalgal wrack (95% *Lobophora* sp.) (experimental treatment 2, CD + MO7) (Table 1). The trial was carried out for 100 days under natural photoperiod and ambient daylight of 1500 lux, and the average rearing water conditions in experimental tanks were as follows: temperature (24.7 ± 0.4°C), dissolved oxygen (6.9 ± 0.1 mg L^−1^), and pH (7.5 ± 0.1).

### 2.3. Growth Parameters, Tissue Collection, and Body Indexes

Fish weight was measured at the beginning, during (monthly), and at the end of the experimental period, and final survival, weight increment, and specific growth rate (SGR: [(ln final weight − ln initial weight)/time] × 100) were determined for each treatment (23 individuals per treatment). At the end of the experiment, five specimens from each diet were starved for 24 h prior to slaughter, and samples of muscle, liver, and digestive tract were collected and immediately stored at −80°C until biochemical analysis. Hepatosomatic (HSI: (liver weight/body weight) × 100), viscerosomatic (VSI: (viscera weight/body weight) × 100), and visceral-fat indexes (VFI) were also determined (*n* = 5). VFI was calculated from visible fat of organs according to the following scale: 1 (low), 2 (medium), or 3 (high) [26].

### 2.4. Proximate and Lipid Composition

The moisture content of diets (CD, CD + MU7, and CD + MO7) and fish muscle (n =5) was obtained by drying in an oven at 110°C to constant weight, while the ash content by dry ashing in a furnace at 450°C for 24 h [27]. Kjeldahl nitrogen analysis was used to determine the crude protein content.

Total lipid (TL) from both MU and MO wracks, diets and *C. idella* muscle (*n* = 5), was extracted according to the Folch method as described by Christie and Han [28]. Briefly, samples were homogenized in 10 mL chloroform/methanol (2 : 1, v/v) using a Virtis rotor homogenizer (Virtishear, Virtis, Gardiner, New York), and 2.5 mL of 0.88% KCl (w/v) was added to the homogenate. After vigorous shaking, samples were centrifuged at 716 × g for 5 min, and the organic solvent was collected, filtered through a filter paper (Filter-Lab, Barcelona, Spain), and evaporated under a stream of nitrogen. The lipid content was determined gravimetrically, resuspended in chloroform/methanol (2 : 1, v/v) with 0.01% (w/v) butylated hydroxytoluene (BHT; Sigma-Aldrich Co., St. Louis, Missouri, USA) at 10 mg mL^−1^, and stored at -20°C under an inert atmosphere of nitrogen.

Lipid classes (LC) were analyzed following Reis et al. [29]. A 30 *μ*g aliquot of TL was developed by high-performance thin-layer chromatography (HPTLC) with a single-dimensional double-development in 10 × 10 cm HPTLC plates (Merck KGaA, Darmstadt, Germany). Polar lipids were separated using 1-propanol/chloroform/methyl acetate/methanol/0.25% KCl (5 : 5 : 5 : 2 : 1.8, v/v), while hexane/diethyl ether/acetic acid (20 : 5 : 0.5, v/v) were used for the neutral lipids. The different LC was visualized by charring at 160°C after spraying with 3% (w/v) aqueous cupric acetate containing 8% (v/v) phosphoric acid and quantified by means of a CAMAG TLC Visualizer (Camag, Muttenz, Switzerland).

Fatty acid methyl esters (FAME) were obtained by acid-catalyzed transmethylation of 1 mg of TL extract. FAME were purified by thin-layer chromatography (TLC) in 20 × 20 cm TLC plates (Macherey-Nagel, Duren, Germany) and separated and quantified using a TRACE-GC Ultra Gas Chromatograph (Thermo Scientific, Milan, Italy). Chromatographic conditions were programmed as previously described by Galindo et al. [19]. Individual FAME were identified by reference to authentic standards (Mix C4-C24 and PUFA No. Three from menhaden oil (Supelco Inc., Bellefonte, Pennsylvania, USA)), and further confirmation of FAME identity was carried out by GC-MS (DSQ II, Thermo Scientific) when necessary.

### 2.5. Total Antioxidant Activity of Macroalgal Wracks

A 25 g sample of MU and MO dry seaweed wrack was successively extracted three times with *n-*hexane, ethyl acetate, and ethanol (250 mL each × 24 hours) by maceration at room temperature with continuous stirring, and the filtered solution was concentrated in vacuum to obtain the extracts (Table S1, Supplementary Material). Dried extracts and a standard solution of Trolox were dissolved in sterile dimethyl sulfoxide (DMSO) using a sonication bath for 3–4 min at a final concentration of 50 mg mL^−1^ [20].

Antioxidant activity was evaluated by the 2,2'-Azino-bis(3-ethylbenzothiazoline-6-sulfonic acid) (ABTS) radical scavenging assay [30]. Reaction was prepared by mixing 7 mM ABTS (w/v) solution and 2.4 mM potassium persulfate solution (w/v) for 12-16 h at room temperature in the dark. Resultant solution was then diluted in methanol in order to obtain an absorbance of 0.7 at 734 nm. Serial dilutions of extracts in methanol were carried out in 96-well microplates, with concentrations ranging between 0.244 and 250 *μ*g mL^−1^. A control was also prepared in each plate. ABTS solution was added to the microwells, and after 8 min of incubation, the absorbance was recorded at 750 nm with a BioRad Microplate Reader Model 680 (Bio-Rad Laboratories, Inc., Hercules, CA, USA). The same procedure was applied to the standard solution of Trolox (0.098-100 *μ*g mL^−1^).

The 1,1-diphenyl-2-picryl-hydrazyl (DPPH) radical scavenging assay was conducted following Blois [31] in 96-well microplates in quadruplicates. Serial dilutions of samples in methanol (0.244-250 *μ*g mL^−1^) and Trolox were tested, with a control being also added to all microplates. DPPH dissolved in methanol (90 *μ*g mL^−1^; w/v) was added to the microwells, and the absorbance was measured at 515 nm, after 30 min in the dark.

The percentage of antioxidant activity (% AA) for ABTS and DPPH assays was calculated by using the formula (1):

(1) $$\begin{array}{c} \%\text{AA}=\left[\frac{\left({\text{Abs}}_{\text{control}}–{\text{Abs}}_{\text{sample}}\right)}{\left({\text{Abs}}_{\text{control}}\right)}\right]\text{ x }100, \end{array}$$

where Abs_control_ is the absorbance of ABTS radical + methanol, and Abs_sample_ is the absorbance of ABTS radical + sample/standard.

Concentration yielding 50% scavenging of ABTS (IC_50_) of each sample was calculated by interpolation from the % AA *vs*. concentration curve.

### 2.6. Antioxidant Response

Antioxidant response of fish was evaluated in five fish per treatment. Peroxide index (PxI) was determined in the muscle of *C. idella* as detailed by Shantha and Decker [32]. Briefly, a 3 mg aliquot of TL was dissolved in 10 mL of chloroform/methanol (7 : 3, v/v) and mixed with 50 *μ*L of ferrous chloride (FeCl_2_) and hydrochloric acid (HCl) solution, together with 50 *μ*L of ammonium thiocyanate (NH_4_SCN). After vigorously shaking, and let soak for 5 min, absorbance was measured at 500 nm. The concentration of lipid peroxides was calculated using a ferric chloride (FeCl_3_) standard curve and expressed as meq O_2_ Kg^−1^.

Prior to the analysis of thiobarbituric acid reactive substances (TBARS) and antioxidant enzymes, 300 mg of each sample was homogenized in an ice-cold 20 mM Tris-HCl (w/v) buffer (pH 7.4) with protease inhibitors (Complete®, Sigma, Madrid, Spain). Muscle and liver samples were centrifuged at 10,000 × g for 5 min, and supernatants were collected and stored at −80°C until analysis.

TBARS assay was used to determine the content of malondialdehyde (MDA) in the samples. A 144 *μ*L sample homogenate was incubated for 60 min at 95°C in a solution containing 36 *μ*L of 8.1% (w/v) sodium dodecyl sulfate (SDS) with 0.05% (w/v) BHT, 270 *μ*L of 20% (v/v) acetic acid (pH 3.5), and 270 *μ*L of 0.8% (w/v) thiobarbituric acid [33]. At the end of this period, samples were cooled in ice, mixed with 180 *μ*L of Milli-Q water together with 900 *μ*L of n-butanol/pryridine (15 : 1, v/v), and centrifuged at 10,000 × g for 3 min at 4°C. Finally, 200 *μ*L of supernatant was fluorimetric determined with excitation at 530 nm and emission at 550 nm in a multiwell plate reader (Thermo Scientific Appliskan, Thermo Fisher Scientific, Vantaa, Finland). MDA content was calculated using a standard curve of tetramethoxipropane (TMP) and expressed as nmol MDA mg protein^−1^.

Among antioxidant enzymes assayed, SOD activity was measured as described earlier by Mesa-Herrera et al. [34], using a reaction buffer (pH 8.2) containing 50 mM Tris-cacodylic acid, 1 mM DTPA, and 30 mM pyrogallol as substrates. The absorbance was read at 420 nm. One unit of SOD activity is equivalent to the amount of enzyme that produces a 50% inhibition of the autooxidation of pyrogallol.

CAT enzymatic activity was analyzed according to Claiborne [35], using hydrogen peroxide (H_2_O_2_) 485 mM (v/v) as substrate and 10 mM KH_2_PO_4_-10 mM K_2_HPO_4_ solution as reaction buffer (pH 7). Degradation of H_2_O_2_ was measured at 240 nm. The molar extinction coefficient of H_2_O_2_ used for the calculations was 42.6 M^−1^ cm^−1^.

GR activity was measured using a 0.1 M NaH_2_PO_4_-Na_2_HPO_4_ reaction buffer (pH 7.0), with 1 mM GSSG and 60 *μ*M NADPH as substrates [36]. Oxidation of NADPH was determined at 340 nm. The molar extinction coefficient used was -6.22 mM^−1^ cm^−1^.

GST reaction was developed in 0.1 M phosphate buffer (pH 6.5) with 5 mM GSH and 1 mM 1-chloro-2,4-dinitrobenzene (CDNB) as substrates [37] and measured at 340 nm. The molar extinction coefficient of the conjugated between GSH and CDNB formed in the reaction (GS-DNB) was 9.6 mM^−1^ cm^−1^.

One unit of activity was defined as *μ*mol minute^−1^ for all antioxidant activities unless otherwise stated.

### 2.7. Digestive Enzymes

The activity of pancreatic enzymes was determined in five fish per treatment according to Solovyev et al. [38] in order to prevent sample deterioration. Prior to analysis, gut samples were homogenized in 10 volumes (v/w) of ice-cold Milli-Q water, centrifuged at 3,300 × g for 3 min at 4°C, and supernatants kept at -80°C until enzymatic quantification.

Alkaline protease activity was determined as described by García-Carreño and Haard [39]. Briefly, samples were incubated at 24°C for 60 min using azocasein (0.5%) in Tris-HCl 50 nmol L^−1^ (pH 9) as substrate, and the reaction stopped with 20% trichloroacetic acid (TCA). After centrifugation at 10,000 × g for 5 min, absorbance of the supernatant was read at 366 nm. One unit of activity (U) was defined as 1 *μ*mol of azo dye released per min per mL.

Alpha-amylase (E.C. 3.2.1.1) was assayed using 0.3% soluble starch dissolved in Na_2_HPO_4_ buffer (pH 7.4) according to Métais and Bieth [40]. The absorbance was measured at 580 nm after stopping the reaction with 1 N HCl and the addition of 2 mL of N/3000 iodine solution (Merck, Darmstadt, Germany). Alpha-amylase activity (U) was defined as the mg of starch hydrolysed at 37°C per 30 min per mL.

Bile salt-activated lipase (BAL, E.C. 3.1.1) activity was analyzed by incubation with p-nitrophenyl myristate dissolved in 0.25 mM Tris-HCl, pH 9.0, 0.25 mM 2-methoxyethanol, and 5 mM sodium cholate buffer for 30 min at 30°C. Acetone/n-heptane (5 : 2, v/v) was used to stop the reaction. Samples were then centrifuged at 6,000 × g for 2 min at 4°C, and the increase in absorbance of the supernatant was determined at 405 nm [41]. BAL activity (U) corresponded to the *μ*mol of myristate hydrolysed per min per mL.

Absorbances were measured in a spectrophotometer (Beckman Coulter DU800 Fullerton, California, USA). Soluble protein of homogenized samples was quantified following Bradford [42], using bovine serum albumin as standard. Specific activity is expressed as mU and U mg protein^−1^.

### 2.8. Statistical Analysis

Prior to analysis, normality and homocedasticity were confirmed within groups and when necessary, data were transformed using logarithm or arcsine square root. Differences between treatments were assessed by one-way ANOVA followed by the Tukey HSD post hoc test. Welch test followed by the Dunnett T3 was used for no homoscedastic data, and Kruskall-Wallis nonparametric test was applied in the case of no normal distribution followed by pair-wise comparison Mann–Whitney test with Bonferroni correction.

Results are presented as mean ± standard deviation (SD), and the statistical significance was set at *P* < 0.05. All statistical analyses were performed using IBM® SPSS Statistics 25.0 software package (IBM Corp., New York, USA) for Windows.

## 3. Results

### 3.1. Proximate Composition and Fatty Acid Profile of Macroalgal Wracks and Diets

The TL content of *Lobophora* sp. MO wrack (3.49% dry weight (DW)) was higher than that of the MU wrack (2.51% DW) (Table 1). Both wracks were characterized by a high proportion of SFA (39-47%), chiefly 16:0 (28-36%). Although both total n-6 PUFA and n-3 PUFA were more abundant in the MO wrack (19.93% and 14.14%, respectively) than in the MU one (8.66% and 6.27%, respectively), the n-3/n-6 ratio remained unchanged at around 0.7. Total n-3 LC-PUFA was higher in the MO wrack, mainly due to its 4-fold higher EPA content, whereas DHA remained reduced in both seaweed wracks (0.29% in MU and 0.43% in MO).

As shown in Table 1, moisture, protein, and lipid proportions were similar in the three diets (10-13%, 34-36%, and 9% DW, respectively), whereas ash content was slightly lower in the CD than in diets supplemented with 7% of either macroalgal wrack (7 *vs*. ~10% DW, respectively).

A 7% inclusion of macroalgae did not affect dietary FA composition. Thus, total MUFA was the main group of FAs (40-41%) in all diets, mainly due to C18 isomers (~34%) followed by SFA which represented near a quarter of total FAs, with 16:0 being the most abundant component (16%). Both total n-6 and total n-3 PUFA were also similar in the three diets (~22% and 10-11%, respectively). Regardless of EPA and DHA variations between wracks, their proportions in the experimental diets remained unchanged and similar to those of the CD (Table 1).

### 3.2. Survival, Growth Parameters, and Body Indexes

Survival was greater than 80% in all treatments. Preliminary studies performed by our group with *C. idella* juveniles showed a detriment in growth with an inclusion of 20% of the same MU tested in the present work (data not shown). By contrast, a 7% dietary inclusion of either MU or MO tended to improve fish growth parameters with respect to the control treatment; although, these differences were not significant. Additionally, *C. idella* juveniles fed CD + MU7 presented the healthiest VSI and VFI values (Table 2).

### 3.3. Proximate and Lipid Composition of Muscle

The proximate composition of muscle from *C. idella* juveniles was not affected by dietary composition (Table 2). Thus, moisture (79-80%), protein (77-82% DW), ash (5-6% DW), and TL (6-9% DW) remained unchanged in all fish groups.

Regardless of dietary treatment, muscle LC profile was characterized by a higher proportion of total neutral lipids (TNL) (55-65% of TL) than total polar lipids (TPL) (Table 3). TNL were mainly represented by triacylglycerols (TAG), which accounted for 31-43% and cholesterol (COL) with 11-15% of TL, whereas phosphatidylcholine (PC) was the main phospholipid in all fish groups (17-23%), followed by phosphatidylethanolamine (PE) (10-11%) and phosphatidylserine + phosphatidylinositol (PS + PI) (5-7%). Differences between dietary treatments were only significant for sterol esters (SE), which was lower in the CD-fish (3.10 ± 0.71%) than in the experimental fish (4.5-5.9%) (Table 3).

Muscle FA composition was also similar between groups, except total n-6 PUFA, which was higher in CD+7MU-fish (18.13 ± 1.27%) than in CD-fish (15.73 ± 1.15%) (Table 4). Total MUFA was the most abundant family of FA with 38-43% of total FAs, mainly due to 18:1, followed by SFA (25-26% of total FA), with 16:0 as its main component (18-19%). DHA was the greatest n-3 PUFA in muscle, ranging from 6.55 ± 2.88% in CD+7MO-fish to 8.60 ± 3.07% in CD+7MU-fish, while relative amounts of EPA were notably lower (2.31-2.70% of total FA).

### 3.4. Total Antioxidant Activity of Macroalgal Wracks

Total antioxidant capacity of MU and MO wracks is displayed in Table 5. Every extract from MU wrack showed low activity for DPPH (<40%). Both ethyl acetate and ethanol extracts from MO wrack scavenged the DPPH radical by more than 50% at 250 *μ*g mL^−1^, with ethyl acetate surpassing 80%. For these two samples, the IC_50_ values were calculated. The most active extract (ethyl acetate) was near 10-fold less active than Trolox (IC_50_ values of 70.80 *μ*g mL^−1^*vs*. 7.43 *μ*g mL^−1^).

All extracts except that of ethanol from MU wrack exceeded 50% activity after the ABTS assay. Overall, MO wrack extracts were more active than MU ones. Ethyl acetate extract from MO wrack was the most active of all extracts, with an IC_50_ of 13.17 *μ*g mL^−1^, over 15-fold higher than that of Trolox (0.87 *μ*g mL^−1^).

### 3.5. Antioxidant Activities and Lipid Peroxidation

**Figure 1:**
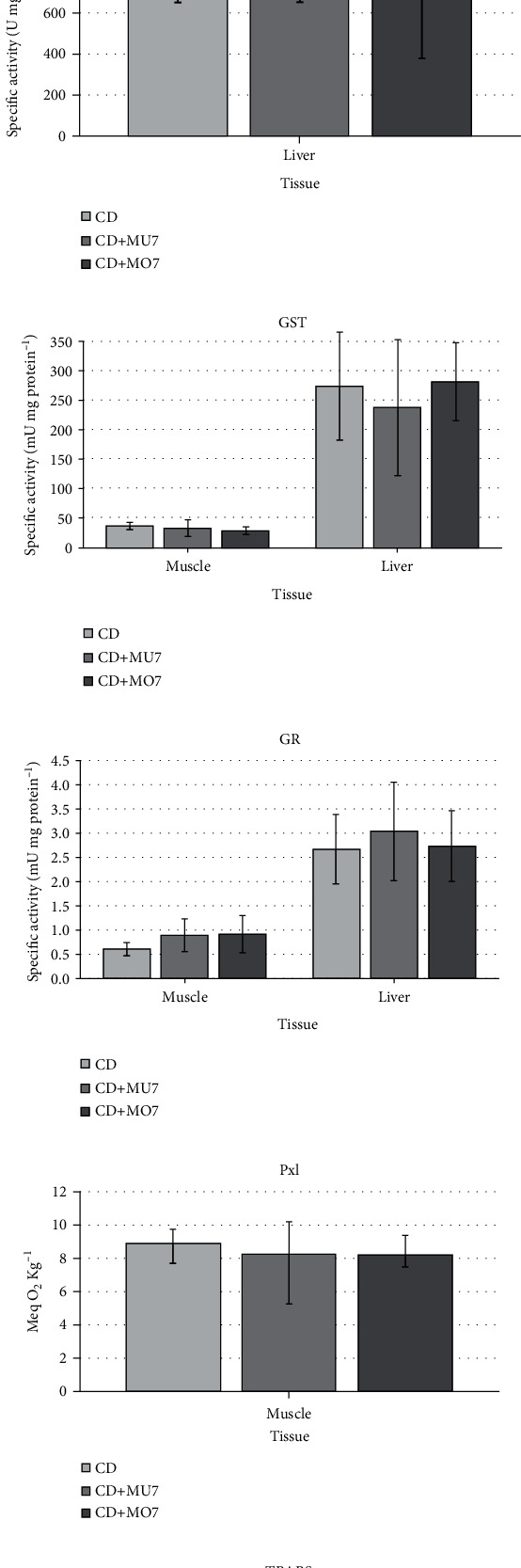
Antioxidant activities: (a) catalase (CAT), (b) superoxide dismutase (SOD), (c) glutathione-S-transferase (GST), (d) glutathione reductase (GR), (e) peroxides index (PxI), and (f) TBARS. CAT, GST, GR, and TBARS were determined in the muscle and liver, SOD was determined in the liver, and PxI was determined in the muscle from *C. idella* juveniles fed the different diets. Results are presented as mean ± SD (*n* = 5). CD: control diet; CD + MU7: control diet supplemented with 7% multispecific macroalgal wrack; CD + MO7: control diet supplemented with 7% monospecific macroalgal wrack. Different letters denote significant differences among dietary treatments (*P* < 0.05).

CAT, GST, and GR activities were determined in the muscle and liver, and SOD was determined in the liver from *C. idella* juveniles (Figure 1). All antioxidant activities were higher in the liver than in the muscle. None of the activities differed between treatments, except that of CAT, which was near the half in the liver of both experimental fish groups (12-13 U mg protein^−1^) than in the control one (26 U mg protein^−1^).

Finally, PxI and TBARS were determined in order to assess the oxidative status of the fish. PxI remained unchanged in the muscle, ranging between 8 and 9 meq O_2_ Kg lipid^−1^, whereas TBARS varied between 0.3 and 0.6 nmol MDA mg protein^−1^ in the muscle and 1-1.2 nmol MDA mg protein^−1^ in the liver.

### 3.6. Digestive Enzymes

The activity of the intestine digestive enzymes (Table 6) did not differ between fish groups. Thus, alkaline protease activity varied between 186 and 256 mU mg protein^−1^, BAL activity was 13-34 mU mg protein^−1^, and that of alpha-amylase ranged between 167 and 289 U mg protein^−1^.

## 4. Discussion

Marine macroalgae have been proposed as a valuable alternative to terrestrial plants in aquafeed formulation, not only for their potential as protein-nutritive sources but also because of their bioactive-compounds that make them potential candidates to promote fish health and welfare. However, most investigations have been focused on a reduced number of carnivorous fish species where *Ulva* (Chlorophyta), *Gracilaria*, and *Porphyra* (Rhodophyta) genus are the most studied seaweeds due to their high availability and commercial value. By contrast, dietary inclusion of brown macroalgae has been little studied [2, 8, 43, 44].

The inclusion of seaweed and microalgae in fish diets has been shown to improve some growth performance parameters [45]. Thus, weight gain of both *Dicentrarchus labrax* and *S. aurata* was enhanced with a 5 and 10% dietary inclusion of *Pterocladia capillacea* [9, 46], respectively. These two species also showed a greater growth rate with a 5% inclusion of *Ulva* sp. [9, 46]. Moreover, Chen et al. [47] demonstrated that a low inclusion (5%) of *Chlorella sorokiniana* improved *Oncorhynchus mykiss* growth, while a 10% inclusion did not exert any effect. By contrast, a 10% inclusion of *Ulva lactuca* or *Enteromorpha linza* resulted in lower growth rates in *O. mykiss* juveniles compared to the control diet [48]. These contradictory results might be attributed to the different digestibility of macroalgae species included in fish diets that may change depending on their level and type of complex polysaccharides that can act as barriers and chelators, hampering their digestion. Nonetheless, herbivorous and omnivorous fish have been shown to better digest dietary seaweeds [45].

In our study, a dietary 7% inclusion of either a mixture of seaweed wracks species (MU) or a *Lobophora* sp. wrack (MO) produced similar growth performance than the commercial control diet. The utilization of seaweeds as an ingredient *per se*, at an industrial scale, requires expensive processes that makes it often not economically viable. Thereby, in the last years, the interest for macroalgae has emphasized on a quality more than on a quantity approach, based on their content in bioactive compounds. Hence, the inclusion of macroalgae in aquafeeds is now more focused on low inclusion levels, than on their use as main ingredients [8].

*O. mykiss* showed a reduction in viscerosomatic and hepatosomatic indices with the dietary inclusion of >25% of the microalgae *A. platensis* [49]. In our study, the reduction of both indices with the 7% inclusion of MU could be attributed to the presence of the carotenoid fucoxanthin in brown algae such as *Lobophora* sp. and *Dictyota* sp., which were part of the MU wrack. Fucoxanthin has been described to present antiobesity and lipolytic effects [50]. The possible synergetic effect between macroalgal species in the MU wrack could be favoring a stronger lipolytic action than *Lobophora* sp. alone (MO wrack). Furthermore, it seems that bioavailability (solubility and adsorption) of fucoxanthin in humans may be affected by the copresence of certain lipids [51]. Hence, the better performance of MU over MO may be also related to some favorable combination of lipids in the multispecific wrack. A reduction in VSI may be an economic advantage for fish production as viscera is often discarded; so, lower VSI decreases the volume of by-product produced. Additionally, reduced HSI indicate no negative impact of seaweed inclusion on fish health [49].

Dietary inclusion of macroalgae has been described to vary muscle proximate composition of fish. Thus, the high protein content of macroalgae meals has been related to higher muscle content, whereas fish fed diets supplemented with *Ulva* sp. reduced their lipid content in the muscle [8]. In our study, the absence of differences in the proximate composition of *C. idella* muscle could be probably associated with the use of isoproteic and isolipidic diets.

Both qualitative and quantitative effects of macroalgae meal on lipid metabolism have been previously reported although the mechanism has yet to be fully elucidated [6, 8]. In accordance to our experiment, dietary FA profiles are generally reflected in fish muscle FA composition [8]. However, MU inclusion seems to slightly enhance a selective retention of n-6 PUFA. N-3 PUFA was reported to be selectively retained in the muscle from *Solea senegalensis* fed with a 5% of *Ulva ohnoi* dietary inclusion [8].

Overall, muscle LC were not affected by the dietary treatment, except SE, which were more abundant in both groups of fish receiving macroalgae. SE are formed from sterol in a normal homeostatic process that can be enhanced after an excess of sterol ingestion. They are also a storage form of FA [52]. Marine macroalgae are rich in phytosterols [19], including several molecules such as fucosterol, stigmasterol, sitosterol, and saringosterol, together with variable amounts of COL [53]. The higher contribution of phytosterols in both diets containing macroalgae may be enhancing the SE synthesis/deposition by the fish. It is important to stress that phytosterols are known to lower total and low-density lipoprotein cholesterol levels in humans [54].

The total antioxidant capacity of MO and MU wracks used in our work showed promising results. Contrarily to the DPPH assay, all MO and MU extracts, except MU-ethanol, were capable of inhibiting more than 50% of the radicals at 250 *μ*g mL^−1^ in the ABTS assay. The greater sensitivity of the ABTS method compared to that of DPPH would explain these results [20]. All extracts from the wrack mainly formed by *Lobophora* sp. had the highest antioxidant activities. Ito et al. [55] reported a higher DPPH activity in *Ochrophyta* species than in species from other phyla, attributing it to their content of polyphenols and tocopherols. Furthermore, fucoxanthin is a typical pigment from some *Lobophora* species, in particular *Lobophora variegata* [56]. Several beneficial properties have been attributed to fucoxanthin, including the free radical scavenging and single oxygen species quenching, resulting in antioxidant capacities [57]. Besides fucoxanthin, antioxidant molecules present in brown algae comprise other pigments, phlorotannins, sulphated polysaccharides, and sterols [20]. The antioxidant activity detected in macroalgal wracks is essential since it can potentially contribute to the protection of protein and lipids, among others, in fish [20]. Nonetheless, results from *in vitro* studies assessing macroalgae antioxidant capacity can be hard to extrapolate to complex living organisms [58].

Understanding the effects of seaweed supplementation in fish is particularly important in aquaculture because farming protocols often induce stress conditions that affect the immune system responses, including an increase in ROS production that might compromise growth performance and animal welfare [14]. The antioxidant capacity of macroalgal wracks studied here did not affect SOD, GST, or GR activities in neither liver nor muscle from *C. idella* juveniles. However, CAT activity was decreased in the liver, but not in the muscle of fish fed macroalgae. The decrease in antioxidant activities has been suggested to be an indicator of a reduced requirement to remove hydrogen peroxide and lipid peroxides from tissues [43]. However, this was not reflected in the oxidative status of muscle or liver. Thus, MDA content (TBARS) and PxI are products of PUFA peroxidation that can be used as biomarkers of oxidative damage [59, 60]. Although no significant differences were found in PxI and MDA contents, TBARs tended to be lower in muscle of CD+7MU-fish. Chen et al. [47] showed that a 10% inclusion of *C. sorokiniana* significantly decreased MDA content in both kidney and liver of *O. mykiss*, while a 5% of inclusion did not have any effect, suggesting that the absence of significant differences in our study may be due to the percentage of inclusion chosen.

Feed composition, and, in particular, the inclusion of algae in diets have demonstrated to influence the activity of enzymes involved in digestive and absorptive processes in several fish species [43, 44]. However, these effects depend on factors such as the species, the dietary inclusion level, type of seaweed, duration of feeding, mode of supplementation, and even rearing conditions [43]. Thus, the inclusion of *Gracilaria pygmaea* decreased protease activity in *O. mykiss* [43], probably due to the presence of antinutritional factors such as protease inhibitors [43]. The absence of changes in protease activity in our experimental fish suggests a reduced content of antinutrients in the seaweeds used or an insufficient level of dietary inclusion. Similarly, neither lipase nor amylase activities were affected by the diets. Lipase is known to have higher preference for PUFA as substrate [43]. The similar dietary PUFA contribution in our experimental design might also be related with the absence of differences in the BAL activity with algal inclusion. Overall, dietary algal inclusion did not affect digestive enzymes activities, suggesting the capacity of *C. idella* juveniles to adapt to changes in dietary composition, and as a consequence, to obtain similar growth rates in all experimental groups.

In conclusion, the macroalgal wracks from Gran Canaria coasts used in the present work might be considered as a potential feed additive for fish. Thus, a 7% of macroalgae may be included in the diet without detrimental effect on *C. idella* survival, growth, proximate composition, FA or LC profile, oxidative status, and digestive capacity. Although antioxidant activity would depend on the relative abundance of macroalgae species in the collected wracks and their conservation status, the MU wrack enhanced protective activity of CAT in the liver, leading to a lower and healthier perivisceral fat deposition. Limitations such as composition stability of seaweed wracks should be taken into account for their potential use in aquaculture. Despite the financial feasibility of the proposed activity should be also deeply analyzed, the utilization of macroalgal biomass that is usually discarded in aquafeeds might contribute to the sustainable use of ocean resources empowering the blue economy strategy in islands, also reducing aquaculture reliance on fish oil and fish meal.

## Figures and Tables

**Table 1:** Proximate composition (moisture, protein, ash, and total lipid content), total fatty acids, and main fatty acid composition of multispecific (MU) and monospecific (MO) wracks and diets used in the experiment.

	MU wrack	MO wrack	CD^∗^	CD^∗^ + MU7	CD^∗^ + MO7
Moisture (%)	—	—	12.89	10.22	12.49
Protein (% of DW)	—	—	35.63	34.25	35.20
Ash (% of DW)	—	—	7.30	10.49	10.09
Total lipid (% of DW)	2.51	3.49	9.19	8.92	9.28
Total FA (*μ*g mg DW^−1^)	—	—	70.95	71.71	75.16
*Fatty acids (% of total FA)*					
Total SFA	47.18	39.34	24.00	23.79	23.76
14:0	6.09	7.83	2.23	2.29	2.28
16:0	36.31	28.17	16.16	16.03	15.97
18:0	2.00	1.00	4.11	4.01	4.03
Total MUFA	32.47	21.66	41.01	40.38	40.33
16:1^1^	12.02	6.99	4.37	4.49	4.44
18:1^2^	19.61	13.94	34.50	33.83	33.94
20:1^2^	nd	nd	0.95	0.93	0.94
Total n-6 PUFA	8.66	19.93	22.48	22.32	22.31
18:2	2.79	9.73	21.38	21.35	21.35
20:4	4.23	7.31	0.70	0.74	0.74
Total n-3 PUFA	6.27	14.14	10.41	11.21	10.96
18:3	0.99	1.49	3.80	3.88	4.03
20:5	2.46	8.42	3.14	3.46	3.30
22:5	nd	nd	0.53	0.47	0.43
22:6	0.29	0.43	2.26	2.47	2.28
n-3/n-6	0.72	0.71	0.46	0.50	0.49
Total n-3 LC-PUFA	3.19	8.96	6.11	6.65	6.19

DW: dry weight; CD: control diet; CD + MU7: control diet supplemented with 7% multispecific macroalgal wrack; CD + MO7: control diet supplemented with 7% monospecific macroalgal wrack; FA: fatty acid; SFA: saturated fatty acids; MUFA: monounsaturated fatty acids; PUFA: polyunsaturated fatty acids; LC-PUFA: long-chain polyunsaturated fatty acids (≥C20 and ≥2 double bonds). Totals include other minor components not shown. ^1^Mainly n-7 isomer for diets and n-9 for macroalgal wracks; ^2^mainly n-9 isomer. nd: not detected. ^∗^Ingredients: corn gluten feed, wheat, processed animal proteins from poultry, soya meal feed, fish meal, and rapeseed oil.

**Table 2:** Growth parameters, body indexes, and muscle proximate composition of *C. idella* juveniles fed the experimental diets.

	CD	CD + MU7	CD + MO7
Growth parameters			
Weight increment (g)	16.96 ± 7.23	24.30 ± 9.45	19.95 ± 8.79
SGR (% day^−1^)	0.39 ± 0.13	0.75 ± 0.24	0.45 ± 0.19
Body indexes			
HSI (%)	1.47 ± 0.40	1.30 ± 0.51	1.54 ± 0.62
VSI (%)	7.99 ± 1.25^b^	6.85 ± 0.87^a^	7.74 ± 1.42^ab^
VFI	2.80 ± 0.41^b^	1.93 ± 0.70^a^	2.67 ± 0.49^b^
Proximate composition			
Moisture (%)	79.68 ± 3.38	79.50 ± 1.32	79.00 ± 1.76
Protein (% DW)	81.63 ± 6.31	78.38 ± 7.19	77.26 ± 8.65
Ash (% DW)	5.48 ± 0.71	6.07 ± 1.12	6.05 ± 0.96
Total lipid (% DW)	6.47 ± 0.84	7.31 ± 2.28	8.51 ± 1.89

Results are presented as mean ± SD (*n* = 3 for growth parameters; *n* = 5 for body indexes and proximate composition). CD: control diet; CD + MU7: control diet supplemented with 7% multispecific macroalgal wrack; CD + MO7: control diet supplemented with 7% monospecific macroalgal wrack; SGR: specific growth rate; HSI: hepatosomatic index; VSI: viscerosomatic index; VFI: visceral-fat index; DW: dry weight. Different letters in superscript in the same row denote significant differences among dietary treatments (*P* < 0.05).

**Table 3:** Lipid class composition of the muscle from *C. idella* juveniles fed the different diets.

Lipid class (% of total lipid)	CD	CD + MU7	CD + MO7
SM	1.74 ± 1.10	2.61 ± 1.38	1.22 ± 0.46
PC	22.65 ± 3.14	20.26 ± 3.07	16.61 ± 4.69
PS + PI	6.49 ± 0.94	6.56 ± 1.46	5.12 ± 1.27
PG	2.30 ± 0.39	1.97 ± 0.59	1.94 ± 0.48
PE	11.42 ± 2.57	11.39 ± 3.59	10.22 ± 3.02
TPL	44.60 ± 3.85	42.14 ± 7.76	35.11 ± 9.13
MAG	2.50 ± 0.42	1.65 ± 0.29	2.59 ± 0.76
DAG	1.47 ± 0.55	1.52 ± 0.29	0.96 ± 0.33
COL	12.88 ± 1.50	15.07 ± 2.94	11.14 ± 2.40
FFA	2.88 ± 0.85	2.44 ± 0.82	2.47 ± 0.53
TAG	32.58 ± 6.51	30.68 ± 10.97	43.18 ± 11.80
SE	3.10 ± 0.71^a^	5.86 ± 1.50^b^	4.55 ± 0.61^b^
TNL	55.40 ± 3.85	57.86 ± 7.76	64.89 ± 9.13

Results are presented as mean ± SD (*n* = 5). CD: control diet; CD + MU7: control diet supplemented with 7% multispecific macroalgal wrack; CD + MO7: control diet supplemented with 7% monospecific macroalgal wrack; SM: sphingomyelin; PC: phosphatidylcholine; PS: phosphatidylserine; PI: phosphatidylinositol; PG: phosphatidylglycerol; PE: phosphatidylethanolamine; TPL: total polar lipids; MAG: monoacylglycerols; DAG: diacylglycerols; COL: cholesterol; FFA: free fatty acids; TAG: triacylglycerols; SE: sterol esters; TNL: total neutral lipids. Different letters in superscript within the same row indicate significant differences between treatments (*P* < 0.05).

**Table 4:** Total fatty acids and main fatty acid composition of the muscle from *C. idella* juveniles fed the different diets.

	CD	CD + MU7	CD + MO7
Total FA (mg FA 100 g wet weight^−1^)	939.63 ± 156.75	1096.37 ± 387.10	1145.12 ± 312.46
*Fatty acids (% of total FA)*			
Total SFA	25.88 ± 0.92	25.38 ± 1.34	25.33 ± 0.94
14:0	1.86 ± 0.33	1.47 ± 0.39	1.85 ± 0.34
16:0	18.87 ± 0.61	18.44 ± 0.79	18.70 ± 0.58
18:0	4.75 ± 0.77	4.81 ± 1.02	4.32 ± 0.82
Total MUFA	42.07 ± 5.82	38.27 ± 6.57	42.89 ± 5.99
16:1^1^	8.16 ± 1.43	6.02 ± 1.02	7.82 ± 1.44
18:1^2^	34.17 ± 4.39	31.04 ± 5.36	33.81 ± 4.46
20:1^2^	0.92 ± 0.37	0.97 ± 0.12	1.02 ± 0.11
Total n-6 PUFA	15.73 ± 1.15^a^	18.13 ± 1.27^b^	16.12 ± 1.53^ab^
18:2	9.90 ± 0.79	10.84 ± 1.77	10.58 ± 1.16
20:4	3.67 ± 1.18	4.83 ± 1.53	3.43 ± 1.59
Total n-3 PUFA	12.36 ± 3.56	14.49 ± 3.47	11.92 ± 3.61
18:3	1.83 ± 0.31	1.66 ± 0.43	1.77 ± 0.28
20:5	2.31 ± 0.71	2.70 ± 0.44	2.38 ± 0.76
22:5	0.90 ± 0.20	1.10 ± 0.19	0.89 ± 0.20
22:6	6.84 ± 2.41	8.60 ± 3.07	6.55 ± 2.88
n-3/n-6	0.78 ± 0.18	0.79 ± 0.14	0.73 ± 0.15
Total n-3 LC-PUFA	10.52 ± 3.42	12.83 ± 3.63	10.16 ± 3.85

Results are presented as mean ± SD (*n* = 5). CD: control diet; CD + MU7: control diet supplemented with 7% multispecific macroalgal wrack; CD + MO7: control diet supplemented with 7% monospecific macroalgal wrack; FA: fatty acid; SFA: saturated fatty acids; MUFA: monounsaturated fatty acids; PUFA: polyunsaturated fatty acids; LC-PUFA: long-chain polyunsaturated fatty acids (≥C20 and ≥2 double bonds). Totals include other minor components not shown. ^1^Mainly n-7 isomer; ^2^mainly n-9 isomer. Different letters in superscript denote significant differences among dietary treatments (*P* < 0.05).

**Table 5:** DPPH and ABTS activity and IC_50_ of multispecific (MU) and monospecific (MO) macroalgal wracks used in the experiment.

		DPPH		ABTS	
		Activity (%)	IC_50_ (*μ*g mL^−1^)	Activity (%)	IC_50_ (*μ*g mL^−1^)
MU wrack	*n-*Hexane	12.12 ± 2.47^a,^^∗^	>250	53.24 ± 5.03^b,^^∗^	197.04 ± 11.96^∗^
	Ethyl acetate	38.37 ± 1.66^b,^^∗^	>250	79.03 ± 1.57^c^	69.89 ± 1.56^∗^
	Ethanol	7.04 ± 0.57^a,^^∗^	>250	27.82 ± 2.14^a,^^∗^	>250
MO wrack	*n*-Hexane	24.42 ± 2.15^A^	>250	74.08 ± 0.96^A^	79.83 ± 3.70^C^
	Ethyl acetate	89.63 ± 2.91^C^	70.80 ± 1.06^A^	78.99 ± 0.95^B^	13.17 ± 0.68^A^
	Ethanol	62.53 ± 1.68^B^	169.91 ± 4.11^B^	80.20 ± 1.72^B^	43.87 ± 1.36^B^
Trolox		92.21 ± 0.32	7.43 ± 0.74	83.25 ± 1.28	0.87 ± 0.18

Results are presented as mean ± SD. All determinations were carried out by quadruplicate. DPPH: 1,1-diphenyl-2-picryl-hydrazyl; ABTS: 2,20-Azinobis-(3-ethylbenzothiazoline-6-sulfonic acid); IC_50_: concentration yielding 50% scavenging of each radical.^a,^^b,^^c,^ and ^A,^^B,^^C^ represent significant differences between solvents within the same wrack (*P* < 0.05). ^∗^ Represents significant differences between wracks for the same solvent (*P* < 0.05). Activity (%) was measured at 250 *μ*g mL^−1^ for MU and MO extracts and at 100 *μ*g mL^−1^ for Trolox standard.

**Table 6:** Digestive enzymes activity determined in *C. idella* juveniles fed the different diets.

	CD	CD + MU7	CD + MO7
Alkaline proteases (mU mg protein^−1^)	226.00 ± 94.49	185.94 ± 44.27	255.61 ± 155.16
Bile salt-activated lipase (mU mg protein^−1^)	25.59 ± 14.26	34.22 ± 21.86	13.35 ± 7.92
Alpha-amylase (U mg protein^−1^)	167.21 ± 78.04	289.34 ± 120.90	223.19 ± 46.30

Results are presented as mean ± SD (*n* = 5). CD: control diet; CD + MU7: control diet supplemented with 7% multispecific macroalgal wrack; CD + MO7: control diet supplemented with 7% monospecific macroalgal wrack.

## Data Availability

The authors declare that the data used to support the findings of this study are included within the article.

## Supplementary Materials

Supplementary Materials Table S1: percentage yield of crude extracts obtained from macroalgal wrack by maceration.
