# Supplementary material for: Valorization of Seaweed Wracks: Inclusion as Additive in Diets for Grass Carp (Ctenopharyngodon idella)
Source: Aquac Nutr. 2022 Dec 5;2022:6992682. doi: 10.1155/2022/6992682 (PMC9973163; doi:10.1155/2022/6992682)
Supplement: Supplementary Materials — Table S1: percentage yield of crude extracts obtained from macroalgal wrack by maceration. [file 6992682.f1.docx]

**SUPPLEMENTARY MATERIAL**

**Inclusion of seaweed wracks in diets for grass carp (*Ctenopharyngodon idella*). Effects on growth performance, muscle lipid profile, antioxidant status and digestive capacity**

Ana Galindo^1^, Covadonga Rodríguez^1^, Diana B. Reis^1^, Manuel Marrero^1^, Nieves G. Acosta^1^, Maria Carmo Barreto^2^, Ignacio. A. Jiménez^3^, Jaime de Urioste^4^, Marianna Venuleo^5^, José A. Pérez^1*^

^1^ Departamento de Biología Animal, Edafología y Geología, Universidad de La Laguna. Avenida Astrofísico Francisco Sánchez s/n, 38206 La Laguna, Tenerife, Spain

^2^ cE3c–Centre for Ecology, Evolution and Environmental Changes/Azorean Biodiversity Group and Faculty of Sciences and Technology, University of Azores, 9500-321 Ponta Delgada, Portugal

^3^ Instituto Universitario de Bio-Orgánica Antonio González, Departamento de Química Orgánica, Universidad de La Laguna. Avenida Astrofísico Francisco Sánchez 2, 38206 La Laguna, Tenerife, Spain

^4^ Centro de Investigación y Conservación de la Biodiversidad, Fundación Neotrópico. C/ Piñonero 9, 38311 Barranco Grande, Tenerife, Spain

^5^ Departamento de Biotecnología, División de Investigación y Desarrollo Tecnológico, Instituto Tecnológico de Canarias. Playa de Pozo Izquierdo, s/n, 35119 Santa Lucía de Tirajana, Gran Canaria, Spain

*Correspondence: janperez@ull.edu.es; Tel.: 0034 922318340

**Table S1**. Percentage yield of crude extracts obtained from macroalgal wrack by maceration.

| Macroalgal wrack | Macroalgal weigth (g) | Solvent extraction | Extract weigth (g) | Percentage yield (% DW) |
| --- | --- | --- | --- | --- |
| Multispecific | 25 | *n-*hexane | 0.44 | 1.91 |
|  | 25 | Ethyl acetate | 0.20 | 0.87 |
|  | 25 | Ethanol | 1.28 | 5.58 |
| Monospecific | 25 | *n*-hexane | 0.64 | 2.87 |
|  | 25 | Ethyl acetate | 0.57 | 2.56 |
|  | 25 | Ethanol | 3.45 | 15.38 |

DW, Dry weight
